# Sexual coercion and health-risk behaviors among urban Chinese high school students

**DOI:** 10.3402/gha.v7.24418

**Published:** 2014-05-14

**Authors:** Yi Song, Cheng-Ye Ji, Anette Agardh

**Affiliations:** 1 Department of Child, Adolescent and Women’s Health, Institute of Child and Adolescent Health, School of Public Health, Peking University, Beijing, China; 2 Division of Social Medicine and Global Health, Department of Clinical Sciences, Lund University, Lund, Sweden

**Keywords:** risk behaviors, sexual coercion, high school students, violence

## Abstract

**Objective:**

To determine the association between health-risk behaviors and a history of sexual coercion among urban Chinese high school students.

**Design:**

A cross-sectional study was performed among 109,754 high school students who participated in the 2005 Chinese Youth Risk Behavior Survey. Data were analyzed for 5,215 students who had experienced sexual intercourse (1,483 girls, 3,732 boys). Multivariate logistic regression was used to determine the relationship between sexual coercion and the related covariates, and data were stratified by gender.

**Results:**

Of those students who had had sexual intercourse, 40.9% of the females and 29.6% of the males experienced sexual coercion (*p*<0.01). When analyses controlled for demographic characteristics, in the study sample, that is, students who had sexual intercourse, drug use (odds ratios [OR], 2.44), attempted suicide (OR, 2.30), physical abuse (OR, 1.74), binge drinking (OR, 1.62), verbal abuse (OR, 1.29), experience of being drunk (OR, 0.68), and smoking of cigarettes (OR, 0.52) were related to a history of sexual coercion. Patterns of health-risk behaviors also differed among female and male students who had experienced sexual coercion.

**Conclusions:**

Sexual coercion is associated with health-risk behaviors. Initiatives to reduce the harm associated with sexual coercion among high school students are needed.

Sexual coercion is a public health concern that may have numerous adverse health consequences, including rape victimization, unintended pregnancy, and sexually transmitted diseases (STDs) and HIV/AIDS (1–5). Globally, the prevalence of sexual coercion varies greatly between countries and regions. In the United States, the National Violence Against Women Survey (NVAWS) estimated that one in six women and one in thirty-three men have experienced an attempted or completed rape at some point in their lifetime, and that the majority of first rape victims (both females and males) are under age 18 (6). Findings from the 2003 Youth Risk Behavior Surveillance (YRBS) conducted in the United States show that 11.9% of female and 6.1% of male students in grades 9–12 had at some time forced sexual intercourse (7). The prevalence of sexual coercion varies from 5 to 50% in less developed countries such as northern Thailand (males 6.5%, females 21.0%) (8), Kenya (males 11.0%, females 20.8%) (9), Uganda (male students 29.0%, female students 33.1%) (10), and Peru, where almost half of the young women and a quarter of the young men reported sexual coercion (11). Even in China, where the prevalence of sexual intercourse is very low among high school students (4.8%), the prevalence of forced sex is 39.2% among those students who had previously engaged in sexual behavior. The prevalence of sexual coercion among girls (40.9%) was higher than boys (29.5%) (12). Results across settings, despite the variety of methods used or differences in sampling, consistently find a greater prevalence of sexual coercion perpetrated against women, although both female and male youths are vulnerable.

Sexual coercion has been shown to be associated with a number of risky behaviors that in turn may also have adverse health consequences. For example, such coercion is significantly associated with early sexual debut, having multiple sexual partners, and inconsistent condom use (4, 10). Some studies also found that patterns of risk behaviors differed among sexually active male and female adolescents who reported being forced or pressured to have sex (13, 14). The results from a study of 3,931 girls and 3,953 boys in the United States found that having been forced or pressured to have sex was associated with externalizing behavior such as fighting among girls, and with internalizing behavior such as bulimia among boys (14).

Several researchers have also confirmed an association between sexual coercion and poor mental health, including suicidal ideation (15, 16). Feeling sad or hopeless, having considered or attempted suicide, being a victim of physical dating violence, heavy cigarette use, binge drinking, having multiple sexual partners, and engaging in unprotected sex were associated with a history of sexual coercion (16). A study conducted in 132 schools in the United States by Raghavan et al. (17) determined that the first sexual experiences of many victimized young female adolescents may have been forced. Moreover, young women who have been sexually assaulted may try to alleviate their emotional distress with alcohol and drug use, which can impair their judgment and reduce the ability to escape from dangerous situations (17). In a study by Agardh et al., exposure to violence was significantly associated with the experience of sexual coercion among both males and females, and sexual coercion and threats of violence were significantly associated with poor mental health (15).

In China, one hospital-based study of adolescent women also indicated that sexual coercion was more likely to be associated with health-risk behaviors (e.g. alcohol abuse or multiple sex partners), being beaten or abused by one's partner, younger age at first sexual intercourse, prostitution, and exchanging material benefits for sex (18). A local study in Henan province similarly found that the sexual abuse of Chinese girls was associated with increased rates of depression, sadness, suicidal thinking and planning, and health-related risk behaviors such as drinking alcohol, smoking, fighting, and having sexual intercourse (19). Many researchers have advocated promoting sexual negotiation, contraceptive use, and STD/HIV prevention among adolescents (20, 21). Moreover, China has a large youth population (22.0% between the ages of 10 and 24 years in 2011) (22), and a 9-year compulsory educational system that would enable a potential intervention regarding safe sex to be conducted in schools for most young people.

However, little is known about the prevalence of sexual coercion among adolescents in China. Research into sexual coercion using national samples is rare, and the extent to which adolescents have been forced to have sexual intercourse is still not known. The first nationwide survey, the 2005 Chinese Youth Risk Behavior Survey (CYRBS), was designed to assess six categories of priority health-risk behaviors that contribute to the leading causes of death, disability, and social problems in China in order to estimate the prevalence and epidemic trend of those behaviors among adolescents.

The CYRBS forms the basis of the current investigation of the association between sexual coercion and other health-risk behaviors. To the best of our knowledge, the present study is the first to use data from a national sample to investigate sexual coercion among urban Chinese high school students. Its objective is to describe the prevalence of sexual coercion in different populations and to determine the association between sexual coercion and such health-risk behaviors as suicidal ideation, physical violence, substance abuse, and runaway behavior.

## Methods

### Population and setting

The study was conducted in urban high schools in 18 provinces of China: Beijing, Tianjin, Hebei, Liaoning, Heilongjiang, Shanghai, Jiangsu, Fujian, Jiangxi, Henan, Hubei, Hunan, Guangdong, Guangxi, Hainan, Sichuang, Qinghai and Xinjiang. A total of 109,754 high school students in grades 10–12 participated in the 2005 CYRBS, among which 5,215 students (4.8% of all participants) reported that they had had sexual intercourse, and 1,706 students (1.55%) reported that they had experienced sexual coercion. Among these 1,706 students who reported sexual coercion, two had not had sexual intercourse, and they were omitted from further analysis. We focused our study on the 5,215 students who reported a history of having had sexual intercourse, as they would be better able to answer whether they had been forced to this or not than students with no experience of sexual intercourse. Such a strategy reduces any potential ambiguity with regard to how high school students might perceive sexual coercion, defined here as forced sexual intercourse. Thus, among these 5,215 students, 1,704 had experience of sexual coercion, 3,497 had no such experience, and 14 cases were missing. The mean age of this sample was 17.3±1.2 (range 14–20); 1,483 were girls (28.4%) and 3,732 were boys (71.6%). A description of the age, grade, type of school, family structure, and socioeconomic status of the students is provided in Table 1.

**Table 1:** Characteristics of Chinese high school students who had had sexual intercourse

Demographics	All (*n*=5215) *n* (%)	Girls (*n*=1,483) *n* (%)	Boys (*n*=3,732) *n* (%)
Age			
14	12 (0.2)	5 (0.3)	7 (0.2)
15	244 (4.7)	91 (6.1)	153 (4.1)
16	1,123 (21.5)	348 (23.5)	775 (20.8)
17	1.762 (33.8)	542 (36.5)	1,220 (32.7)
18	1,419 (27.2)	363 (24.5)	1,056 (28.3)
19	454 (8.7)	93 (6.3)	361 (9.7)
20	201 (3.9)	41 (2.8)	160 (4.3)
Mean (±SD)	17.3±1.2	17.1±1.1	17.3±1.2
Grade			
10	1,890 (36.2)	520 (35.1)	1,370 (36.7)
11	1,929 (37.0)	555 (37.4)	1,374 (36.8)
12	1,396 (26.8)	408 (27.5)	998 (26.5)
School type			
Key school	1,313 (25.2)	353 (23.8)	960 (25.7)
Ordinary school	1,507 (28.9)	361 (24.4)	1,146 (30.7)
Vocational school	2,390 (45.9)	767 (51.8)	1,623 (43.5)
Family structure			
Both parents	4,094 (79.8)	1,114 (76.5)	2,980 (81.1)
Single parent	579 (11.3)	173 (11.9)	406 (11.0)
One biological parent and one step-parent	149 (2.9)	70 (4.8)	79 (2.1)
Grandparents	140 (2.7)	45 (3.1)	95 (2.6)
Other	171 (3.3)	55 (3.8)	116 (3.2)
Socioeconomic status			
High	1,961 (38.2)	619 (42.6)	1,342 (36.5)
Middle	1,800 (35.1)	467 (32.1)	1,333 (36.3)
Low	1,370 (26.7)	368 (25.3)	1,002 (27.3)

### Study design

This study utilizes a cross-sectional design using data obtained from the 2005 CYRBS. The study was designed to describe six categories of priority health-risk behaviors that contribute to the leading causes of death, disability and social problems in China, including a broad range of health-risk behaviors. The survey questionnaire was adapted from the 2003 YRBS in the United States (23), in which a high degree of reliability and validity has been documented by previous studies (24–26). In addition, our questionnaire was reviewed and validated by education and health experts and pilot-tested in Beijing and Jinan (27). The appropriateness and feasibility of the actual survey were assessed in focus groups and through interviews with the teachers and students.

### Data collection

The survey used a three-stage cluster sample design to obtain a nationally representative group of students. At the first sampling stage, three cities or districts (highly-developed, mid-level developed, and developing) from each province were sampled according to their socioeconomic levels. At the second sampling stage, 447 high schools were selected with probability proportional to school enrollment size (PPS sampling). The third sampling stage consisted of randomly selecting one or two classes from grades 10 to 12 at each high school in the survey. All students in the classes selected were eligible to participate. Information was obtained by means of anonymous, self-administered questionnaires that assessed unhealthy diet-related behaviors, physical inactivity, unintentional injuries and violence, substance abuse, internet addiction, and sexual behaviors that may cause unintended pregnancies and STDs. Sociodemographic characteristics, such as sex, grade, family structure, type of school, and socioeconomic status of the household, were obtained from sampling (13). Ethical approval of this study including the consent procedure was obtained from Peking University's Medical Research Ethics Committee. Informed consent was obtained verbally because written consent was difficult to obtain in the large sample. We had the name list of every chosen class, and thus the participants’ consent could be recorded by the investigators who collected the data. All of the respondents were informed that this survey would be conducted anonymously as well as confidentially, and that their privacy would thus be respected. The response rate for lifetime experience of sexual behaviors among all students was 97.4%, and the response rate for lifetime experience of sexual coercion was 99.7% among those students who had previously had sexual intercourse. The overall response rate regarding questions concerning health-risk behaviors was 98.2% among the high school students sampled. The prevalence of sexual intercourse ranged from 1.9 to 7.9% across the different provinces.

### Description of variables

#### Sociodemographic variables

The demographic variables concerning age, grade, family structure, and socioeconomic status were recorded and coded as necessary, based on their frequency distribution and appropriateness for statistical analysis. The schools were divided into three types: ordinary school, key school (those with records of past educational accomplishment were given priority in the assignment of teachers, equipment, and funds, as well as the privilege of recruiting the best students) and vocational schools.

#### Dependent variable

*Experience of sexual coercion* was measured by the question, ‘Have you ever been forced to have sexual intercourse when you did not want to?’ Response categories were ‘Yes’ or ‘No’.

#### Independent variables

*Mental health* included the following three variables: 1) *Feeling sad/hopeless* was measured by the question ‘During the past 12 months, did you ever feel so sad or hopeless almost every day for 2 weeks or more in a row that you stopped doing some usual activities?’ 2) *Considered suicide* was based on the response to the question ‘During the past 12 months, did you ever seriously consider attempting suicide?’ 3) *Attempted suicide* was ascertained by the response to ‘During the past 12 months, did you actually attempt suicide?’ All responses to the above variables were coded as ‘Yes’ or ‘No’.

*Experience of physical violence* encompassed the following three variables: 1) *Physical fight* was measured by the question: ‘During the past 12 months, how many times were you in a physical fight?’ The response alternatives were coded as: ‘0 times’, ‘1 time’, ‘2 times’. 2) *Physical abuse* was based on the response to the question: ‘During the past 30 days, have you ever been hit, slapped, or physically hurt by someone on purpose?’ 3) *Verbal abuse* was ascertained by the response to: ‘During the past 30 days, have you ever been threatened and terrified?’ All responses were coded as ‘Yes’ or ‘No’.

*Substance use* was assessed by the following four questions: 1) *Smoking cigarettes* was measured by the question ‘During the past 30 days, on the days you smoked, how many cigarettes did you smoke per day?’ Response categories were coded as ‘0 cigarettes’, ‘1 or less cigarettes’, ‘≥2 cigarettes’ per day. 2) *Binge drinking* was based on the response to the question ‘During the past 30 days, on how many days did you have five or more drinks of alcohol in a row that is, within a couple of hours?’ Response categories were coded as ‘0 days’, ‘1–5 days’, ‘≥6 days’. 3) *Experience of being drunk* was ascertained by the response to ‘During the past 12 months, how many times did you get drunk (feeling light-headed, headache, sleepy, etc.) because you drank too much?’ 4) *Drug use* was assessed by the question ‘How many times have you used illegal drugs like ice crystal methamphetamine, marijuana, cocaine, heroin, or opiates (medication containing codeine or morphine, etc.)?’ For the questions of *Experience of being drunk* and *Drug use*, if the answer was 0 times, we coded it as ‘No’, and if the answer was 1 time or more, we coded the responses as ‘Yes’.

*Running away from home* was judged by the following two questions: 1) *Considered running away from home* was based on the response to the question ‘During the past 12 months, did you ever seriously consider running away from home, that is, leaving your home for 24 hours or more without your parents’ permission?’ 2) *Attempted running away from home* was measured by the question ‘During the past 12 months, did you ever attempt to run away from home (for 24 hours or more without your parents’ permission)?’ Responses were coded as ‘Yes’ or ‘No’.

### Data analysis

SPSS Statistical Software Version 18.0 was used to perform all data analysis. Multivariate logistic regression analyses were limited to the 4,723 students for which complete information was available, as follows: 14 of the 5,215 records were excluded because information on sexual coercion was missing; an additional 478 records were excluded because information for one or more health-risk behaviors was missing. The overall analytic goal was to evaluate the association between health-risk behaviors and sexual coercion. Multivariate models were used to test the range of health-risk behaviors that were independently associated with sexual coercion. The prevalence of sexual coercion for each independent variable was estimated for the total sample and stratified by gender. Data was first analyzed by using univariate logistic models between the dependent variable and each independent variable. The variables that were found to be significant in the univariate models were then entered together with the demographic variables into a multivariate logistic regression analysis in order to yield the final model. Thus, multivariate analysis was used to determine those health-risk behaviors that were independently associated with sexual coercion. Adjusted odds ratios (OR) and 95% confidence intervals (CI) were examined to assess the significance of the relationships. Significance level was accepted at *p*<0.05, two-tailed.

## Results

### General description of sexual coercion

Among the 5,215 study subjects, 32.8% reported a history of sexual coercion. The reported lifetime prevalence of sexual coercion for girls (40.9%) was approximately 1.4 times higher than for boys (29.5%). The prevalence was greatest for 15-year-old adolescents (42.0%), key school students (36.9%), and youth who lived in a developing city or district (37.4%) (Table 2).

**Table 2:** Prevalence of sexual coercion among students in relation to sociodemographic factors and health-risk behaviors

Risk factors	All (*n*=5,201)%	Girls (*n*=1,482)%	Boys (*n*=3,719)%
Age			
14	33.3	40.0	28.6
15	42.0	58.2	32.2
16	37.6	46.6	33.5
17	33.4	38.6	31.0
18	30.7	37.2	28.5
19	21.9	28.0	20.3
20	28.4	46.3	23.8
Educational level			
Grade 10	33.7	44.5	29.6
Grade 11	33.5	39.8	31.0
Grade 12	30.5	37.8	27.4
School type			
Key school	36.9	45.3	33.9
Ordinary school	32.8	46.0	28.6
Vocational school	30.5	36.4	27.7
Family structure			
Both parents	32.6	41.0	29.4
Single parent	30.8	40.5	26.7
One biological parent and one step-parent	27.2	35.7	19.5
Grandparents	36.0	40.0	34.0
Other	42.9	45.5	41.7
Socioeconomic status			
High	31.2	35.0	29.4
Middle	31.0	42.0	27.2
Low	37.4	49.5	32.9
Mental health			
Feeling sad/hopeless in last 12 months			
Yes	35.6	44.6	31.3
No	31.3	38.9	28.5
Considered suicide in last 12 months			
Yes	39.2	43.0	37.1
No	29.3	38.8	26.3
Attempted suicide in last 12 months			
Yes	58.4	46.9	63.7
No	29.9	40.2	26.0
Physical violence and violence-related behaviors			
Physical fight in last 12 months			
2+ times	33.8	50.0	31.2
1 time	30.9	37.3	28.9
0 times	32.5	39.6	28.1
Physical abuse in last 30 days			
Yes	51.2	57.4	49.5
No	30.0	38.9	26.2
Verbal abuse in last 30 days			
Yes	47.7	55.7	45.3
No	29.9	38.7	26.2
Substance use behaviors			
No. of cigarettes smoked per day in last 30 days			
≥2 cigarettes	26.6	35.6	25.4
1 or less cigarettes	31.5	37.7	29.4
0 cigarettes	37.9	42.7	34.4
Binge drinking in last 30 days			
≥6 days	46.5	57.4	44.1
1–5 days	27.8	28.8	27.6
0 days	32.8	42.9	27.5
Experience of being drunk in last 12 months			
Yes	29.3	35.1	27.4
No	36.0	44.8	31.7
Drug use			
Yes	56.4	57.9	56.0
No	30.1	39.7	26.1
Runaway behavior			
Considered running away from home in last 30 days			
Yes	33.8	40.3	30.6
No	31.9	41.9	28.5
Attempted running away from home in last 30 days			
Yes	38.6	44.7	36.6
No	31.2	40.5	27.4

### Univariate relationships between sexual coercion and risk factors

Overall, sexual coercion was associated with mental health issues, physical and verbal abuse, substance abuse, and attempting to run away from home, in addition to demographic variables other than grade level (Table 3). Among boys, sexual coercion was associated with considering or attempting suicide; physical or verbal abuse; smoking cigarettes; binge drinking; being drunk; drug use; and attempting to run away from home. However, no significant association was found between feelings of sadness or hopelessness and sexual coercion among boys. Those girls who had been in two or more physical altercations were more likely to have a history of sexual coercion. However, among girls sexual coercion was not associated with suicidal thoughts or behaviors, cigarette smoking and running away from home. Family structure was not associated with sexual coercion either among boys or girls (Table 3).

**Table 3:** Univariate logistic regression analysis of associations between sociodemographic factors, mental health, violence, substance use, runaway behavior, and sexual coercion among urban Chinese students (95% CI)

	Total sample	Girls	Boys
Risk factors	OR (95% CI)	OR (95% CI)	OR (95% CI)
Age			
14	1.26 (0.37–4.36)	0.77 (0.12–5.12)	1.28 (0.24–6.89)
15	1.83 (1.23–2.72)	1.62 (0.77–3.40)	1.53 (0.93–2.52)
16	1.52 (1.09–2.11)	1.01 (0.53–1.93)	1.62 (1.09–2.40)
17	1.27 (0.92–1.75)	0.73 (0.39–1.38)	1.44 (0.98–2.12)
18	1.12 (0.81–1.56)	0.69 (0.36–1.31)	1.28 (0.87–1.89)
19	0.71 (0.48–1.03)	0.50 (0.21–0.96)	0.82 (0.52–1.28)
20 (ref)	1.00	1.00	1.00
Educational level			
Grade 10	1.16 (1.00–1.35)	1.32 (1.02–1.72)	1.11 (0.93–1.33)
Grade 11	1.15 (0.99–1.34)	1.09 (0.84–1.42)	1.19 (0.99–1.42)
Grade 12 (ref)	1.00	1.00	1.00
School type			
Key school	1.34 (1.16–1.54)	1.45 (1.12–1.87)	1.34 (1.13–1.59)
Ordinary school	1.11 (0.97–1.28)	1.49 (1.15–1.92)	1.05 (0.88–1.24)
Vocational school (ref)	1.00	1.00	1.00
Family structure			
Both parents (ref)	1.00	1.00	1.00
Single parent	0.92 (0.76–1.11)	0.98 (0.71–1.36)	0.87 (0.69–1.10)
One biological parent and one step-parent	0.78 (0.54–1.12)	0.80 (0.48–1.32)	0.58 (0.33–1.03)
Grandparents	1.16 (0.82–1.66)	0.96 (0.52–1.77)	1.24 (0.80–1.91)
Other	1.56 (1.14–2.13)	1.20 (0.70–2.07)	1.72 (1.18–2.51)
Socioeconomic status			
High	0.76 (0.66–0.88)	0.55 (0.42–0.71)	0.85 (0.71–1.02)
Middle	0.75 (0.65–0.88)	0.74 (0.56–0.97)	0.76 (0.64–0.91)
Low (ref)	1.00	1.00	1.00
Injury-related behavior			
Feeling sad/hopeless in last 12 months			
Yes	1.22 (1.07–1.37)	1.26 (1.02–1.57)	1.15 (0.99–1.34)
No (ref)	1.00	1.00	1.00
Considered suicide in last 12 months			
Yes	1.56 (1.38–1.76)	1.19 (0.96–1.47)	1.65 (1.42–1.92)
No (ref)	1.00	1.00	1.00
Attempted suicide in last 12 months			
Yes	3.28 (2.72–3.96)	1.32 (0.95–1.83)	5.00 (3.97–6.30)
No (ref)	1.00	1.00	1.00
Physical violence and violence-related behaviors			
Physical fight in last 12 months			
≥2 times	1.06 (0.93–1.21)	1.52 (1.14–2.04)	1.17 (0.99–1.35)
1 time	0.93 (0.78–1.10)	0.91 (0.66–1.25)	1.04 (0.84–1.28)
0 times (ref)	1.00	1.00	1.00
Physical abuse in last 30 days			
Yes	2.46 (2.08–2.90)	2.12 (1.49–3.01)	2.76 (2.28–3.34)
No (ref)	1.00	1.00	1.00
Verbal abuse in last 30 days			
Yes	2.13 (1.83–2.49)	2.00 (1.46–2.73)	2.33 (1.95–2.78)
No (ref)	1.00	1.00	1.00
Substance use behaviors			
No. of smoked cigarettes in last 30 days			
≥2 cigarettes	0.59 (0.52–0.68)	0.74 (0.55–1.00)	0.65 (0.55–0.76)
1 or less cigarettes	0.75 (0.64–0.89)	0.81 (0.60–1.10)	0.79 (0.65–0.97)
0 cigarettes (ref)	1.00	1.00	1.00
Binge drinking in last 30 days			
≥6 days	1.78 (1.47–2.14)	1.80 (1.17–2.75)	2.07 (1.68–2.57)
1–5 days	0.79 (0.69–0.90)	0.54 (0.41–0.71)	1.02 (0.86–1.18)
0 days (ref)	1.00	1.00	1.00
Experience of being drunk in last 12 months			
Yes	0.74 (0.66–0.83)	0.67 (0.54–0.83)	0.82 (0.71–0.94)
No (ref)	1.00	1.00	1.00
Drug use			
Yes	3.00 (2.49–3.61)	2.09 (1.37–3.18)	3.60 (2.93–4.44)
No (ref)	1.00	1.00	1.00
Runaway behavior			
Consider running away from home in last 12 months			
Yes	1.09 (0.97–1.23)	0.94 (0.76–1.15)	1.11 (0.96–1.28)
No (ref)	1.00	1.00	1.00
Attempted running away from home in last 12 months			
Yes	1.39 (1.21–1.59)	1.19 (0.91–1.54)	1.53 (1.31–1.80)
No (ref)	1.00	1.00	1.00

### Multivariate relationships between sexual coercion and risk factors

Table 4 presents the adjusted OR with 95% CI for associations between the health-risk behaviors and sexual coercion (adjusted for the confounding factors of age, educational level, school type, family structure, and socioeconomic status). According to the models, adolescents who had attempted suicide, experienced physical abuse, heavy consumption of alcohol (binge drinking on more than six of the past 30 days), and had used drugs were more likely to report having experienced sexual coercion. Similarly to the univariate analyses, adolescents who smoked cigarettes or had been drunk were less likely to report sexual coercion. Runaway behavior was not associated with sexual coercion. Obvious differences were found in the risk behaviors associated with sexual coercion by gender. Among girls (but not among boys), having sad or hopeless feelings was associated with sexual coercion. Girls who had engaged in binge drinking for 1–5 days in the past 30 days were less likely to report sexual coercion. In contrast, among boys (but not among girls), attempted suicide, physical abuse and verbal abuse were associated with sexual coercion. Boys who reported binge drinking were more likely to report sexual coercion, while boys who smoked cigarettes were less likely to report sexual coercion (Table 4).

**Table 4:** Multivariate logistic regression analysis of associations between mental health, violence, substance use, runaway behavior and sexual coercion among urban Chinese students (95% CI)

	Total sample	Girls	Boys
Risk factors	AOR (95% CI)	AOR (95% CI)	AOR (95% CI)
Mental health			
Feeling sad/hopeless in last 12 months			
Yes	1.02 (0.88–1.18)	1.31 (1.04–1.66)	N/S
No (ref)	1.00	1.00	1.00
Considered suicide in last 12 months			
Yes	1.06 (0.90–1.24)	N/S	0.81 (0.66–1.00)
No (ref)	1.00		1.00
Attempted suicide in last 12 months			
Yes	2.30 (1.81–2.93)	N/S	4.12 (3.02–5.62)
No (ref)	1.00		1.00
Physical violence and violence–related behaviors			
Physical fight in last 12 month			
≥2 times	N/S	1.38 (0.99–1.94)	N/S
1 time		0.87 (0.62–1.24)	
0 times (ref)		1.00	
Physical abuse in last 30 days			
Yes	1.74 (1.41–2.15)	1.48 (0.98–2.23)	1.85 (1.45–2.38)
No (ref)	1.00	1.00	1.00
Verbal abuse in last 30 days			
Yes	1.29 (1.06–1.58)	1.44 (0.99–2.09)	1.40 (1.10–1.77)
No (ref)	1.00	1.00	1.00
Substance use behaviors			
No. of smoked cigarettes in last 30 days			
≥2 cigarettes	0.52 (0.44–0.62)	N/S	0.55 (0.45–0.68)
1 or less cigarette	0.70 (0.57–0.85)		0.69 (0.54–0.88)
0 cigarettes (ref)	1.00		1.00
Binge drinking in last 30 days			
≥6 days	1.62 (1.27–2.07)	1.43 (0.86–2.37)	1.63 (1.23–2.16)
1–5 days	0.98 (0.83–1.16)	0.62 (0.45–0.85)	1.17 (0.96–1.43)
0 days (ref)	1.00	1.00	1.00
Experience of being drunk in last 12 months			
Yes	0.68 (0.59–0.79)	0.65 (0.50–0.85)	0.67 (0.56–0.80)
No (ref)	1.00	1.00	1.00
Drug use			
Yes	2.44 (1.93–3.08)	1.88 (1.14–3.09)	2.55 (1.95–3.33)
No (ref)	1.00	1.00	1.00
Runaway behavior			
Considered running away from home in last 12 months			
Yes	N/S	N/S	N/S
No (ref)			
Attempted running away from home in last 12 months			
Yes	1.03 (0.87–1.23)	N/S	0.99 (0.80–1.23)
No (ref)	1.00		1.00

Note: The models in the table were adjusted for age, educational level, school type, family structure, and socioeconomic status among students who had sexual intercourse.

## Discussion

In this representative sample of Chinese students aged 14–20, the experience of sexual coercion was not uncommon, and was reported by both boys and girls. According to our findings, 32.8% of students who had previously had sexual intercourse reported experiencing sexual coercion, with 40.9% of the girls and 29.6% of the boys reporting significant events of sexual coercion. The findings are in accordance with those in the study conducted in Henan province, China, where Liang found that 3.4% of 7,623 urban high school students aged 15–19 years had had sexual intercourse, and of these, 30.9% reported experiencing sexual coercion (28). The prevalence of sexual coercion was 25.5% among boys and 45.1% among girls (28). Our results are also similar to the studies conducted in sub-Saharan Africa, where the proportion of girls who said that they were ‘not willing at all’ at their first sexual experience was 38% in Malawi, 30% in Ghana, 23% in Uganda and 15% in Burkina Faso (29). However, the prevalence appeared to be far higher than observed in national sample in previous studies in the United States, such as the 2003 YRBS (9.0%) and 2001 YRBS (7.7%) (7, 30). The higher rates of sexual coercion found in the current study may possibly be attributable to differing social customs and cultural background. In China, where sex education is not common, sexual intercourse among teenagers is disapproved of by family, society, and even teenagers themselves.

Our study showed that the experience of sexual coercion was associated with risky health behaviors among students of both sexes. However, the patterns of risk behaviors differed among girls and boys who had experienced sexual coercion at some time in the past. Among girls, we found that sexual coercion was associated with feelings of sadness or hopelessness. However, among boys, it was related to more impulsive behaviors, such as having attempted suicide, engaging in physical or verbal abuse, and the heavy use of alcohol. Our results were similar to findings from previous studies demonstrating that exposure to interpersonal violence (physical assault, sexual assault, or witnessing violence) or being sexually victimized increased the risk for post-traumatic stress disorder, depression, and substance abuse or dependence (2, 18, 31). In addition, some studies found that girls with a history of sexual abuse generally engaged in internalizing behaviors and boys in externalizing behaviors (6). We found that sexual coercion among girls often corresponded with feelings of sadness or hopelessness. Boys, on the other hand, tended to exhibit a broader range of health-risk behaviors than girls, and some of these, such as attempting suicide and binge drinking, would be regarded as externalizing behaviors.

Some theoretical models, such as the Health Behavior Model, including Social Cognitive Theory, and the Theory of Planned Behavior, provide possible mechanisms whereby mental distress and low self-esteem may affect sexual risk behavior (32–34). The point was that the self-efficacy model of health behavior might predict that people who are depressed have less capacity to take control over their lives, including areas pertaining to sexual behavior. However, it is important to keep in mind that this is a cross-sectional study and the direction of causality is thus uncertain.

Our study found a weak negative association among girls between binge drinking and sexual coercion, contrary to a number of studies suggesting that girls who engage in binge drink would be more likely to report sexual coercion (35–37). A study by Hines and Straus also showed that the association between binge drinking and partner violence varied across sites, and in some sites, such as Louisiana, USA, a strong negative association was found between binge drinking and partner violence (38). According to Hines and Straus, a possible explanation for this negative association might be the extent to which binge drinking is normative behavior, as it apparently is in many colleges in the United States. In such settings, the association between binge drinking and sexual coercion may be negative or weak (38).

China constitutes the world's largest consumer as well as producer of alcoholic beverages, and alcohol is accessible to both boys and girls in a culture where drinking, even among teenagers, is acceptable in society (39). A study conducted in China among 54,040 students in grades 7–12 found that 51.1% reported having at some time consumed alcohol (males 58.6%, females 44.3%), and 10.3% reported at least one episode of binge drinking (males 14.4%, females 6.6%) (27). Binge drinking is not uncommon among both genders. Normative binge drinking in China might also explain the weaker relationship found between sexual coercion and binge drinking among girls than among boys in the current study. However, some other factors as yet unidentified may have influenced the association among girls, and in this study, binge drinking was also the risk factor to the sexual coercion when compared with the students without sexual intercourse among both genders (data not shown).

Previous studies have found significant correlations between smoking and sexual activity among boys (16, 40–42). However, the present study showed that boys who smoked cigarettes reported less sexual coercion. Perhaps boys who smoke tend to play a more active role in social interactions and may thus be less likely to become victimized. Smoking among teenagers is still disapproved of by adults and society in general in China, and thus boys who smoke may to some extent be more rebellious, which might make them less likely to be victims. By contrast, boys who engaged in binge drinking had greater OR for sexual coercion, which is in accordance with an earlier study by Wilson, in which college men who reported a history of sexual coercion also said they drank a great deal of alcohol (43). Smoking and drinking, although both regarded as risky behaviors, do represent different experiences. Although both may be done in a spirit of rebellion, binge drinking leads to impaired judgment and the inability to perceive when one may be in danger, while smoking does not. Therefore, boys who engage in binge drinking may more readily become victimized while under the influence of alcohol.

In agreement with many previous studies (6, 7, 16, 17), we found the experience of sexual coercion associated with illegal drug use among both boys and girls. However, although several studies concluded that running away from home was a health-risk behavior often associated with several problematic outcomes that included violence and sexual abuse (44, 45), our study found no obvious support for an association between experience of sexual coercion and considering or attempting to run away from home by boys and girls. Thus, one implication of these findings is that efforts to reduce sexual coercion could involve approaches that incorporate the entire family. Moreover, prior studies also confirmed that the students with family support were less likely to experience sexual coercion (12, 46).

Sexual coercion often refers to girls in Chinese traditional thinking, but our findings show that boys could also be victims of sexual coercion. The primary prevention of sexual coercion (i.e. stopping it before it starts) may succeed in curtailing those further negative physical and mental health consequences. The development of treatment programs for students is also needed. Our findings suggest that parents and school health providers should be alert to signs of abnormal behavior among children (especially boys) because such behavior might be an indication of exposure to or risk of sexual coercion. Healthcare workers, school staff, and community-based agencies must work together to prevent sexual coercion and provide support for those who are victims of sexual coercion.

Despite its large national sample and high response rate, our study has several limitations. The sensitive nature of the topic may have led to underreporting of sexual coercion by high school students, and thus to a possible underestimation of those experiences. However, participation was anonymous, and this may have enhanced the willingness of respondents to answer sensitive questions. A possible limitation is that sexual coercion was solely examined in terms of being forced to have sexual intercourse, and other types of forced sexual activity might also have occurred, including those unrelated to sexual intercourse. However, the use of this relatively narrow definition of sexual coercion has the advantage of reducing possible variations in how the concept ‘sexual coercion’ may have been perceived in this relatively young sample. Another limitation is the cross-sectional nature of this study. We were only able to test associations between risky health behaviors and sexual coercion, not causation or the temporal order of the factors, that is, binge drinking might show concurrence with sexual coercion because it could be a reaction to sexual coercion, or it could represent an increased likelihood of being in situation where sexual coercion is likely to occur, or a mixture of both. Moreover, lack of knowledge about the perpetrator(s) of sexual coercion, is another limitation of the study. In addition, the use of aggregate measures for the purpose of analysis may not have fully reflected the frequency or severity of the behaviors being examined. For example, although physical fighting was coded to reflect its frequency, responses were reduced to three categories: ‘0’, ‘1’, ‘2 or more’ times.

## Conclusions

Our study clearly showed an association between health-risk behaviors and sexual coercion among urban Chinese high school students. Prevalence of sexual coercion was higher among students with health-risk behaviors, such as drug use, attempted suicide, physical abuse, verbal abuse and binge drinking. However, getting drunk and smoking cigarettes were associated with a lower prevalence of sexual coercion. We also found that patterns of health-risk behaviors differed between girls and boys who had experience of sexual coercion, and boys tended to exhibit a broader range of health-risk behaviors than girls. As sexual coercion and its adverse health consequences may bring about far-reaching influences in one's life, intervention efforts to promote youth development and discourage health-risk behaviors should be supported, with particular consideration of gender disparity.
